# The Use of Aloe Vera Extract as a Novel Storage Media for the Avulsed Tooth

**Published:** 2014-07

**Authors:** Samaneh Badakhsh, Tahereh Eskandarian, Tahereh Esmaeilpour

**Affiliations:** 1 Department of Pediatric Dentistry, Zanjan University of Medical Sciences, Zanjan, Iran;; 2 Department of Pediatric Dentistry, Shiraz University of Medical Sciences, Shiraz, Iran;; 3 Laboratory of Stem Cell Research, Department of Anatomy, Shiraz University of Medical Sciences, Shiraz, Iran

**Keywords:** Aloe Vera, Avulsed tooth, Cell viability, Egg white, Periodontal ligament

## Abstract

**Background: **Tooth avulsion is one of the most severe dental traumas which most often occur in children. When immediate replantation is not possible, storage in a proper media may lead to a prolonged survival rate. Aloe Vera is a cactus like plant with green, tapered leaves that are filled with a transparent viscous gel. This medicinal plant has significant anti-inflammatory, antioxidant, antibacterial and antifungal effects. The purpose of this study was to assess the effectiveness of different concentrations of Aloe Vera extract compared to DMEM (cell culture medium) and egg white.

**Methods:** The periodontal ligament (PDL) cells were cultured and certain number of cells were treated with Aloe Vera extract (in four different concentrations), egg white and culture media for 1, 3, 6, and 9 hours. Cell viability was determined by using the (3-[4, 5-dimethylthiazolyl-2]-2, 5-diphenyltetrazolium bromide) assay. Moreover, One-way ANOVA and post hoc (LSD) test were used for analyzing the study groups.

**Results: **The results indicate that culture media and Aloe Vera extract (10, 30, and 50% concentration) were statistically similar and significantly preserved more PDL cells compared to other experimental storage media.

**Conclusion: **Aloe Vera 10, 30, and 50% may be recommended as a suitable storage media for avulsed teeth.

## Introduction

Tooth avulsion is one of the most severe dental traumas, which most often occur in children. It is more common in a newly erupted teeth and its prevalence is approximately 1-16% of all traumatic injuries to the permanent dentition.^1^ The prognosis of replanted tooth depends on the existence of viable periodontal ligament cells (PDL).^1^^-^^3^ If immediate replantation is not possible, storing in a proper storage media might be an alternative fact which may lead to better prognosis and prolong the tooth survival. Both the storage media and extra-alveolar duration are major critical factors in determining the final prognosis.^4^^,^^5^ However, the capacity of the storage media in maintaining PDL cells viability has been shown to be a more important factor. Studies demonstrated that an improper transportation media causes PDL cells necrosis and may result in root resorptions.^1^^-^^5^ Cvek et al. reported that all the teeth stored in a dry circumstances for more than one hour have been ankylosed.^6^ Until now, several transporting media such as Milk, saliva, saline, tap water, culture medium, propolis,^7^ egg white,^5^^,^^8^^-^^10^ oral rehydration solution,^11^^,^^12^ green tea^13^ and the commercial ones (i.e. HBSS and Viaspan) have been examined.^7^

Aloe Vera is a member of liliaceae family. This medicinal plant is cactus like with green, tapered leaves that are filled with a transparent viscous gel.^13^ This gelatinous substance contains 96% water and 75 active properties such as vitamins, enzymes, minerals, sugars, salicylic acids, and amino acids. It has been reported that Aloe Vera has significant anti-inflammatory, antioxidant, antibacterial, antifungal and anticarcinogenic activities.^13^^,^^14^ A great wound healing effect has been also reported.^15^^-^^17^

To the best of our knowledge, literature is scarce about the use of Aloe Vera extract as a storage media for avulsed teeth. Due to Aloe Vera’s great properties and its specific activities, it was considered that Aloe Vera might be a suitable storage media and might be helpful in the management of tooth avulsion. Therefore, the present in vitro study aims at evaluating the capacity of Aloe Vera extract in four different concentrations and various time points on human PDL cells viability. 

## Patients and Methods

*Preparation of Periodontal Ligament Cells (PDL)*

PDL cells were obtained from healthy premolars extracted for orthodontic purposes in the same manner as described by similar studies.^4^^,^^6^^,^^18^ The patients did not have any diagnosed systemic disorder and teeth were extracted atraumatically. After three times rinsing with normal saline, the residual blood was washed away. Within the first 30 minutes, they were taken to the laboratory in a transporting media which consisted of Dulbecco’s Modified Eagle Medium (DMEM) (Biosera, South Africa), 10% Fetal Bovine Serum (FBS) (Biosera, South Africa), 1% antimycotic (PAA) and 1% gentamycine (PAA). Under laminar flow hood, the crown of the tooth was taken with sterile forceps and again rinsed three times with Phosphate Buffer Saline (PBS). The periodontal tissue was scrapped from only one third of the middle root surface in order to minimize contamination with gingival and apical tissues. The tissues were cut to small pieces, centrifuged for 5 minutes at 1200rpm, treated with 1ml collagenase type I (4mg/ml) and 1ml dispase (3 mg/ml) and finally incubated for 1 hour at 37ºC (95% air and 5% CO2). The cellular suspension was cultured in culture media containing DMEM, 1% L-glutamin (Sigma), 10% FBS, 1% antimycotic and 1.8% Human AB Serum. They were cultured until adequate number of cells was available. Finally passage 3-4 was used.

*Preparation of Aloe Vera Extract*

As Tudose A et al. described^14^ the external surface of the Aloe Vera leaf was washed and disinfected with 70% ethanol alcohol. Under a sterile hood, the insides gelatinous substance was cut off from the external shield and triturated in order to achieve a homogenous gel. It was filtered through 0.45 µm filter. For preparing different Aloe Vera concentrations (10%, 30%, 50% and 100%), the DMEM was used as the diluting media.

Other media evaluated in this study included egg white, supplemented culture media (as positive control) and the negative control was media-free condition.

*Determination of pH Level and Osmolality*

pH level of each experimented media was obtained using an Orion pH Meter model 720 A (Orion Research, Inc., Boston, MA). Also, osmolality measurements were performed with a Vapro model 5520 Vapor Pressure Osmometer calibrated from 100 to 500 mosm/kg (Wescor, Inc., Logan, UT) (table 1).

**Table 1:** Mean pH level and osmolality value of experimented storage media

**Storage media**	**pH level**	**Osmolality (mosmol)**
DMEM	6.87	310
Aloe 10%	7.15	324
Aloe 30%	6.94	338
Aloe 50%	6.73	349
Aloe 100%	5.21	296
Egg white	7.6-8.9(19)	250(19)

Dulbecco’s Modified Eagle Medium (DMEM). Aloe Vera (Aloe).

*Exposure to Different Media in Different Time Points*

A 96- well plate was considered for each time point of 1 h, 3 h, 6 h and 9 h and 8×10^ 4^ cells were seeded in each well. The plates were incubated overnight (at 37ºC, 95% air and 5% CO2) after which the culture media was removed and each well was treated with 100 µl of the seven different media for the experimental time points.

*Viability Assay*

MTT assay (3-[4, 5-dimethylthiazolyl-2]-2, 5-diphenyltetrazolium bromide) was used for determining the cell viability.^20^ In this way, 150 µl of MTT (20µl/ml) was placed in each well and incubated for another 4 hours. In order to dissolve the formazan crystals, 150 µl of DMSO (dimethyl sulfoxide) (MP Biomed) was then added. Optical density (OD) was measured at 492*n*m with ELISA plate reader. The percentage of viability was calculated through the following formula:

Viability (%)=OD experiment/OD control×100

The study groups were compared through by One-way ANOVA and post hoc (LSD). P<0.05 was considered as statistically significant. 

## Results

**Figure 1:**
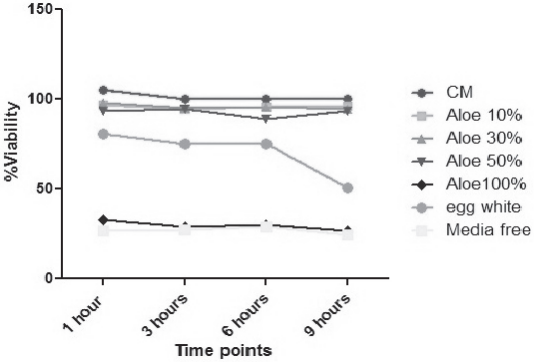
Graph shows the PDL cell viability. CM (Culture Media), Aloe (Aloe Vera).

The capacity of Aloe Vera extract in maintaining the PDL cells viability was assessed by using the MTT assay. As shown in figure 1, supplemented culture media (CM) and Aloe Vera extract in different concentrations (10, 30 and 50%) had the greatest ability to preserve the cell viability at least for up to 9 hours and there was no significant difference among them (table 2). Egg white had a significantly lower capacity compared to Aloe Vera extract (10%, 30% and 50%) and culture media but was significantly better than Aloe Vera 100% and the negative control group. Aloe Vera 100% and the negative control group were similar and were significantly the worst experimental media within the first hour (table 3).

**Table 2:** The P value of the experimented storage media after 9 hours exposure

**Storage media**	**CM**	**Aloe 10%**	**Aloe 30%**	**Aloe 50%**	**Aloe 100%**	**Egg white**
Aloe 10%	0.58					
Aloe 30%	0.643	0.818
Aloe 50%	0.354	0.69	0.856
Aloe 100%	<0.001	<0.001	<0.001	<0.001
Egg white	<0.001	<0.001	<0.001	<0.001	0.003
Media free	<0.001	<0.001	<0.001	<0.001	0.746	<0.001

P<0.05 was considered as statistically significant

**Table 3:** Percentage of viable PDL cells (mean±Std error) for different storage media and time points

**Storage media**	**1 hour** **(mean±SD error)**	**3 hours** **(mean±SD error)**	**6 hours** **(mean±SD error)**	**9 hours** **(mean±SD error)**
DMEM	105±3.07	100±2.09	100±2.52	100±6.23
Aloe Vera 10%	96.98±2.9	94.47±2.23	95.49±3.05	96.27±6.09
Aloe Vera 30%	98.1±2.14	94.86±1.19	97.72±3.07	94.72±8.13
Aloe Vera 50%	93.45±3.5	94.72±2.33	88.92±2.91	93.44±0.82
Aloe Vera 100%	32.88±1.97	29.23±0.48	29.92±1.07	26.67±2.78
Egg white	80.49±4.19	75.37±4.8	75.25±5.52	50.66±13.42
Negative control	26.72±0.54	27.38±0.49	29.03±1.07	24.49±2.84

## Discussion

Presence of viable periodontal ligament cells is the most important factor in determining the final prognosis of an avulsed tooth.^1^^,^^2^^,^^7^ If there is no prospect for immediate replantation performance, the avulsed tooth should be kept in an appropriate storage media in order to prevent the PDL cells death. The transportation media and the time duration between avulsion and replantation are considered as effective factors on the destiny of the replanted tooth. A variety of storage media have been introduced for preserving the PDL cells. Dry storage is remarkably worse than moist media. Andreasen and Andreasen^7^ demonstrated that storing the avulsed tooth in an unsuitable storage media for 45 minutes decreases the success rate to less than 20%. Although in the present study, the negative control group was a media free condition, there was enough humidity in incubator to preserve cell viability in all experimental times (averagely preserving 24.49% viable cells after 9 hours).

Many studies revealed that the cell culture medium (MEM) had the capacity of maintaining the PDL for a long period of time (48-53 hours).^21^ Egg white consists of proteins, vitamins, and water and due to high nutrients value as well as availability in trauma site; it may be considered as a good alternative storage media. In an animal study, Khademi et al. reported that the teeth stored in egg white for 6 to 10 hours were restored more than those kept in milk.^5^ Also, in a distinct in vitro study, it was shown that there were no significant differences between egg white and HBSS and they were superior to tap water and milk.^8^ The findings of a microscopic study by de Sousa et al. demonstrated that after one hour extra-alveolar time, milk and egg white were similar in surviving the PDL cells viability.^9^ Moreover, Rozenfarb et al. observed no significant difference between MEM, egg albumen and milk, although all were superior to saliva.^10^ Based on the result of the present study, egg white may be a suitable storage media for maintaining PDL cell viability. Although approximately 80% of cells survived during the first hour exposure of egg white to PDL cells, counted cells were decreased and half of the cells were averagely viable in the last experimental time point (9 hours). This might be due to reduction of essential nutrients.

Aloe Vera is a natural plant being commonly popular in herbal medicine and nowadays available in many herbal shops.  This plant is well known in wound healing promotion; it has a miraculous healing potency and contains essential nutrients for cells survival.^14^^,^^15^ The inner gel in Aloe Vera leave consists of 96% water and 75 other active components. Historically, many studies have demonstrated that Aloe Vera has superb activities, such as anti-inflammatory, antibacterial, antifungal, anticancer and even antioxidant activities.^13^^-^^16^ Although a number of studies have reported Aloe Vera’s efficacy in dentistry,^15^^,^^22^ literature on the effectiveness of Aloe Vera in preserving PDL cells viability in avulsed teeth is scarce.

The aim of this study was to evaluate the effectiveness of Aloe Vera extract in different concentrations on survival of the PDL cells in an avulsed tooth compared with culture media (supplemented DMEM) and egg white.

Several studies have demonstrated the effectiveness of Aloe Vera extract on wound healing. Davis^17^ reported that wound healing with Aloe Vera was due to the increased blood supply and increased oxygenation which promote fibroblast activity and collagen proliferation.

Results obtained in the present study shows that at least for up to 9 hours supplemented culture media and Aloe Vera extract (in 10, 30, and 50% concentrations) performed similarly and had the greatest capacity in maintaining the cell viability. They could maintain the viability over 90% and were superior to aloe 100% and egg white. In 2009, Tudose and colleagues experimented regenerative capacity of Aloe Vera and compared various plant concentrations of (10, 30, and 50%) at different time exposures (3 h, 6 h, 12, 24 h, and 48 h). Their target cells were human keratinocytes (NCTC2544). Cells treated by Aloe Vera were proliferated compared with  the control group. Investigators showed that there was a gradual increase in cellular growth which was directly correlated with the concentration. It was found that the more concentrated Aloe Vera extract, the more promotion for cellular growth (Aloe Vera 50% compared to 10%). Conclusively, they reported that Aloe Vera has a dose-dependent regenerative potency on skin cells.^14^ In the present study, there were no statistical differences among various concentrations of the Aloe Vera extract (i.e. 10%, 30%, and 50%) with the exception of Aloe Vera 100%.

The negative control group (media free environment) and aloe 100% had the smallest ability in preserving the PDL cells viability. Despite the fact that the 100% concentration of Aloe Vera extract has the most nutrients, there are few probable reasons which may reduce its effectiveness in preserving cell viability. Since pure Aloe Vera is highly viscous, at the time of experiment it entirely covered the surface of the experimented cells and may have possibly prevented the accessibility of oxygen to them. As a result, the more deficiency in oxygenation, the more cell death occurrence. Furthermore, the experiment was carried out on just one layer of attached cells. Consequently, at the time of replacing media, the thickness of aloe 100% may have detached the cells from the floor of the wells. Thus the final counted viable cells were lower compared with the other Aloe Vera’s concentration.

Both pH level and osmolality value of the medium are important and critical criteria for efficacy of the storage media. The optimal cellular growth occurs at an osmolality of 290-300 mosmol/kg (in the range of 230-400 mosmol/kg) and pH level of 7.2-7.4 (in the range of 6.6-7.8).^5^^,^^23^^,^^24^ The pH level of egg white is 7.6-8.9 and its osmolality value is reported about 250 mosmol/kg.^19^ Therefore high nutrients properties and desirable pH level and osmolality of egg white are fully sufficient for being effective in preserving cells viability. As shown in table 1, all experimented concentration of Aloe Vera extract has acceptable osmolality value and pH level with the exception of pure extract of Aloe Vera. The mean pH level of the 100% concentration of Aloe Vera is a bit more acidic (mean pH level=5.21) compared with others. This may be considered as another causative factor for reducing the effectiveness of aloe 100%.

In this study, MTT assay was used for counting the number of the cells. This assay measures the viable cells which are metabolically active and is based on using a yellow tetrazolium salt which changes to insoluble formazan crystal in purple hue. These changes are exclusively performed in metabolically active cells by the action of dehydrogenase enzymes.^20^ Therefore the more brown hue indicates the more viable cells presence. Compared with the traditional methods, this assay is more efficient, more reliable and faster. On the other hand, the Elisa reader device could eliminate intra- and interexaminer variations.

In addition to possessing essential nutrients, Aloe Vera has unique capacity such as antioxidant, antibacterial and antifungal activities. Buttke and Trope suggested that if the storage media has antioxidant ingredients, the efficacy of the media will be improved.^25^ Furthermore, Ozan et al. reported that saliva officinalis is an appropriate storage media due to its antioxidant components and the 2.5% saliva officinalis was superior to HBSS.^26^ Since Aloe Vera has enough antioxidant properties, it is thought to be useful in preserving the PDL cell viability. Martin et al. stated that because of antibacterial and anti-inflammatory activity in propolis, this substance has a high capacity for maintaining the PDL cells.^27^ Moreover, the high success rate of Aloe Vera in protecting the cell viability might be due to its antibacterial and antifungal properties.

Although this in vitro study revealed the effectiveness of the Aloe Vera extract in keeping more viable cells, further research is essential to determine its capacity in reducing or preventing the sequelae which frequently occur following replantation; i.e. root resorptions. 

## Conclusion

Aloe Vera is a natural remedy which is available in many herbal shops and according to the achieved results, aloe 10%, 30% and 50% concentrations may be recommended as a suitable alternative storage media for avulsed teeth. Also, egg white is another accessible storage media in the trauma site and may be considered as an appropriate transporting medium. Further studies are required for definitive confirmation. 
